# Fixed versus Flexible Gonadotropin Releasing Hormone Antagonist Protocol in Controlled Ovarian Stimulation for Invitro Fertilization in Women with Polycystic Ovary Syndrome 

**Published:** 2015-09

**Authors:** Batool Hossein Rashidi, Tahereh Behrouzi Lak, Ensiyeh ShahrokhTehrani, Fatemeh Davari Tanha

**Affiliations:** 1 Reproductive Health Research Center, Tehran University of Medical Sciences, Tehran, Iran; 2 Reproductive Health Research Center, Department of Infertility, Urmia University of Medical Sciences, Urmia, Iran; 3 Department of Obstetrics and Gynecology, MohebeYas Hospital, Tehran University of Medical Sciences, Tehran, Iran

**Keywords:** GnRH Antagonist, Fixed Protocol, Flexible Protocol, PCOS, IVF/ICSI

## Abstract

**Objective:** This study was conducted to compare the results of fixed versus flexible GnRH antagonist protocols in controlled ovarian stimulation for Intra Cytoplasmic Sperm Injection (ICSI) in patients with PCOS.

**Materials and methods:** A randomized clinical trial was performed on 100 PCOS women, who were admitted to a tertiary infertility clinic and were candidate for IVF/ICSI. They were divided into two groups based on the GnRH antagonist protocol. We started GnRH antagonist 0.25mg in flexible protocol when a follicle ≥ 14 mm in diameter was seen in transvaginalsonography (Group 1). In fixed protocol, GnRH antagonist was administered from day 6 of stimulation (Group 2). Number of oocytes in methapase 2, number of developed and frozen embryo as main outcome and days of stimulation, number of gonadotropin and antagonist used assecondry outcome measures were assessed and compared between the two groups.

**Results:** The days of stimulation and the number of antagonist used was not significantly different between fixed and flexible group (p ≥ 0.05).Although the number of gonadotropin injections was significantly lower in flexible group (p = 0.03), the number of oocyte retrieved and the number of embryo which cryopreserved was significantly higher in flexible compared to fixed protocol (p < 0.01).

**Conclusion:** It seems using flexible antagonist protocol in PCOS infertile patients is in favor of better outcomes in terms of number of good quality oocytes and embryo and possibility for cryopreservation for future cycles.

## Introduction

Polycystic ovary syndrome (PCOS) is a common endocrinopathy that affects 5–10% of women of reproductive age (1). These patients present medically complex cases, and are challenging to manage and be treated successfully , due to high rate of cancellation and increased risk of ovarian hyper stimulation syndrome OHSS (2). Despite the development of universally accepted criteria for the diagnosis of the syndrome, the optimal infertility treatment for these patients contains a lot of controversial subjects (3, 4).

Gonadotropin-releasing hormone (GnRH) antagonists are used to prevent luteinizing hormone (LH) surge during controlled ovarian stimulation (COS) without the hypo-estrogenic side-effects, flare-up, or long term down-regulation caused by agonists. They can directly and rapidly inhibit gonadotropin release through competitive attach to pituitary GnRH receptors. This property allows their use at any time during the follicular phase (5).

The pregnancy rates of ART cycles using antagonist may be as good as those achieved by agonists. The beneficial effects like lowering the consumption of FSH ampules, shortening the stimulation period, and minimizing the risk of OHSS would justify a change from the standard long agonist protocol to antagonist regimens (6).

During the last decades, large number of studies confirmed that the introduction of GnRH antagonists provided an effective, safe and convenient alternative to the GnRH agonists (7, 8).

After the introduction, identification of the most appropriate time to start GnRH antagonist administration has been the subject of several studies. The most common type of treatment called fixed protocol in which antagonist started on the 6^th^ day of stimulation with gonadotropins.

However, to reduce the number of antagonist injections and the duration of stimulation, flexible protocol was introduced (9, 10).

Although the GnRH antagonist protocol has been widely used around the world, it is not so popular in some infertility centers, that is mainly due to cost of the drug as well as the lack of practical experiences among infertility specialist .Therefore, the majority of the studies, focused on administrating antagonist in PCOS patients in order to reduce OHSS occurrence (11).

This study was performed to compare the results of fixed versus flexible GnRH antagonist protocols in controlled ovarian stimulation for ICSI in a sub group of infertile patients with polycystic ovary syndrome.

## Materials and methods

One hundred patients with PCOS who were candidate for IVF/ICSI enrolled in this randomized controlled trial, in a tertiary university based infertility clinic between Dec 2012 and Sept 2013. 

They were allocated randomly to different treatments.Women with PCOS diagnosis according to Rotterdam criteria aged between 20-40 yearswere included. Patients with endometriosis, ovarian cyst, thyroid and prolactin disorders, FSH≥12mIU/ml in 3^th^ day of menstruation were excluded. 

Midwife in the clinic recorded the baseline information like age, duration of the marriage, type of infertility, BMI, and lab tests including serum prolactin, FSH, LH, and thyroid tests in prepared questionnaire. A gynecologist performed vaginal ultrasonography (US), in order to assess the ovaries, the number of antral follicles, endometrial thickness and presence of any structural anomaly. 

At the 3^rd^ day of cycle, the patients were randomly (Random Digit Software) allocated to two equal sized groups. 

In flexible group (Group 1), recombinant FSH(Gonal F, Merck-Serono, Switzerland) was started as 150 IU/day, then Cetrotide(Merck-Serono Germany) 0.25 mg was added if the US monitoring showed atleast one follicles with diameter ≥ 14 mm,

On the 3^rd^ day of menstrual cycle in fixed group (Group 2), recombinant FSH 150 IU/day was started, and on the 6^th^ days of the cycle, Cetrotide0.25 mg daily was added into daily injections. 

In both groups 10000 unit of HCG (Pregnyl, Merck & Co, Canada.) was injected when at least 3 follicles with size ≥ 17 mm was seen, and then we performed ovarian puncture after 36-38 hours. ICSI procedure was done, and 2-3 embryos were transferred on day 3 of embryo culture in both groups by the same protocol. The surplus embryos were cryopreserved by vitrification technic at 6-8 cells stage.

Two weeks after embryo transfer, serum βHCG was measured and if the test was positive, progesterone was continued until 8^th^ week. The clinical pregnancy was confirmed by presence of gestational sac with fetal heart rate in ultrasonography in weeks 6-8.Follow up was done until 12 weeks of gestational age. 

Informed consent was obtained from all patients. The study was approved by local Ethical Committee of Tehran University and was registered in clinical trials registry by code number of IRCT 201101135181N5.

The data were analyzed by SPSS using descriptive statistics including independent sample t-test for quantitative and chi-square test for qualitative variables. The P values less than 0.05 were considered statistically significant.

## Results

Table 1 shows the demographic and hormonal characteristics of patients in two groups. As depicted in this table two groups were comparable regarding the age, duration of infertility BMI, hormonal profile and type of infertility (p > 0.05). 

The comparison between the outcome of COS in flexible and fixed groups is shown in table 2. There were significant differences between the two groups regarding mean dose of gonadotropin injections (19.2 ± 4.7, 21.7 ± 6.3; p < 0.05), number of total oocytes (14.75 ± 7.9, 6.9 ± 3.3; p < 0.01), number of oocytes in metaphase II (11.3 ± 6.1, 4.5 ± 2.5; p < 0.01), 2PN fertilized oocytes (8.4 ± 5.1, 4.25 ± 2.3; p < 0.01) and the number of cryopreserved embryo (9.2 ± 4.55, 1.6 ± 2.4; p < 0.01). 

There was no significant difference regarding the mean days of stimulation (9.6 ± 2.3, 10.5 ± 1.2; p = 0.05), dose of antagonist injections (3.8 ± 1.5, 4.7 ± 3.2; p > 0.05 ) , oocytes in metaphase I (1.26 ± 1.98, 1.3±1.1; p> 0.05), germinal vesicle (1.8 ± 2.4, 1.5 ± 1.2; p > 0.05), atretic oocyte (0.5 ± 1.1, 0.12 ± 0.4; p > 0.05),transferred embryo (3 ± 1.23, 2.7 ± 1.1; p > 0.05 ) ,and embryo grading (Grade A: 77.5 % vs. 73.9% ) among patients in flexible vs. fixed group (p > 0.05) (Table 2).

Interestingly the number of 2PN fertilized oocyte and the number of cryopreserved embryo were significantly higher in flexible protocol comparing to fixed protocol (p < 0.01).

In terms of pregnancy outcomes, table 3 shows that chemical and clinical pregnancy rates, as well as, abortion rates were comparable in two groups (p > 0.05).

In women using flexible protocol with reported pregnancy sac, the pregnancy rate after the 12^th^ week was 66.7% (10). In fixed group with reported pregnancy sac there was 100% (18) ongoing pregnancy (p = 0.013) (Table 3).

**Table 1:** Comparison of demographic and hormonal features of patients in Flexible vs. Fixed treated groups

**Variable**	**Group 1** **Flexible (n = 50)**	**Group 2** **Fixed (n = 50)**	**p value**
Age (year)	29.3 ± 4.97	29.5 ± 4.5	NS
Duration of marriage (year)	7.9 ± 4.61	7.2 ± 3.14	NS
Duration of infertility (year)	7.2 ± 4.4	6.5 ± 2.98	NS
Infertility type			
Primary	39 (78)	42 (84)	NS
Secondary	11 (22)	8 (16)	NS
BMI (Kg/m^2^)	25.3 ± 4.9	24.9 ± 2.4	NS
Prolactin(ng/ml)	28.14 ± 66.14	34.26 ± 23.23	NS
TSH (mIU/ml)	2.87 ± 2.44	3.05 ± 1.43	NS
FSH (mIU/ml)	6.63 ± 4	6.45 ± 2.73	NS
LH (mIU/ml)	8.15 ± 5.90	8.28 ± 2.96	NS

Data are presented as Mean ± SD, and Number (%)

**Table 2:** Comparison of COS outcomes in fixed/flexible groups

**Variable**	**Group 1** **Flexible (n = 50)**	**Group 2** **Fixed (n = 50)**	**p value**
Days of ovulation induction (n)	9.6 ± 2.3	10.5 ± 1.2	0.05
Gonadotropin injections (n)	19.2 ± 4.7	21.7 ± 6.3	0.03
Cetrotide injections (n)	3.8 ± 1.5	4.7 ± 3.2	0.07
Oocytes (n)	14.75 ± 7.9	6.9 ± 3.3	< 0.01
Oocytes in metaphase II (n)	11.3 ± 6.1	4.5 ± 2.5	< 0.01
Germinal vesicle	1.8 ± 2.4	1.5 ± 1.2	NS
Atretic	0.5 ± 1.1	0.12 ± 0.4	NS
2PN Fertilized oocyte	8.4 ± 5.1	4.25 ± 2.3	< 0.01
Transferred embryo (n)	3 ± 1.23	2.7 ± 1.1	NS
Frozen Embryo (n)	9.2 ± 4.55	1.6 ± 2.4	< 0.01
Embryo grading			
A	31 (77.5)	34 (73.9)	NS
B	8 (20.0)	10 (21.7)	NS
C	1 (2.5)	2 (4.3)	NS

Data are presented as Mean ± SD, and Number (%)

**Table 3:** Comparison of pregnancy outcomes between the two groups

	**Group 1** **Flexible (n = 50)**	**Group 2** **Fixed (n = 50)**	**p value**
Clinical pregnancy	15 (93.8)	18 (94.7)	NS
Ongoing pregnancy	10 (66.7)	18 (100)	0.01
Early pregnancy loss	1 (6.2)	1(5.3)	NS

Data are presented as number (%)

## Discussion

This study compared ICSI outcomes in flexible GnRH antagonist vs. fixed protocols among patients with polycystic ovary syndrome.

Historically introduction of the GnRH antagonist in COS comes back to about two decades ago. After that, a large number of studies were performed to prove the effectiveness and efficacy of the antagonist comparing to agonist.

Since 2002, following initial Cochrane meta-analysis on the use of GnRH analogues in IVF by Al-Inany et.al, several studies have been published comparing the two GnRH analogues in the general as well as in special groups of patients, like poor ovarian response and PCOS patients (12).

Most recent systematic review and meta-analysis indicate that both analogues are comparable in terms of IVF/ICSI outcomes, except in OHSS occurrence that is higher in agonist protocol (5, 12, 13).

For PCOS patients retrospective and meta-analysis of RCTs findings showed similar stimulation outcomes in agonist and antagonist protocols. However, for severe OHSS, a GnRH antagonist protocol is significantly better in PCOS patients (2, 14).

After a while investigators were interested in comparing the IVF/ICSI outcome regarding to the type of antagonist protocol. So a large number of studies was done to compare fixed versus flexible protocol in different group of patients. 

In kolibianakis study, comparing the fixed and flexible protocol in an RCT found that in flexible regime the antagonist consumption was higher, but no significant difference in total dose of gonadotropin was shown (9). On the contrary, our findings showed that gonadotropin consumption was significantly lower in flexible protocol.

Regarding to the number of oocyte retrieved our results was similar to Ludwig et.al study which showed more oocyte in flexible protocol. (15), but was not in accordance with mochtar et.al study (16).

Several studies have raised concerns regarding an unfavorable effect of late administration of GnRH antagonist, either on day 6 of stimulation or later in flexible protocols (17).

Some researcher suggested that, because a significant proportion of LH surges reported occurred before antagonist initiation, it is worth to evaluate the antagonist administration earlier than day 6 of stimulation (18).

In a randomized controlled trial, they showed that starting the GnRH antagonist either on stimulation day 1 or on stimulation day 6 resulted in equal number of follicular development. In this study, they suggested its use in PCOS patients with high LH levels, during the follicular phase (19).

Recently Hamdine et al in an RCT showed that early initiation of GnRH antagonist results in a more stable endocrine profile, with more physiological levels of E2 and LH during the follicular phase. They recommended establishing larger trails for evaluation of the effect on clinical outcomes (20).

We found that biochemical pregnancy rates was not statistically different in fixed vs. flexible GnRH antagonist protocols among PCOS patients , which was in accordance to Kolibianakis’s study (9).

In our study, despite the trends towards a higher ongoing pregnancy rate in patients with fixed protocol, it seems that the sample sizes of the study is too small to detect the differences of pregnancy outcomes.

Escudero et al conducted an RCT to compare the efficacy of two protocols of multiple dose GnRH antagonists. Days needed for ovarian stimulation were similar in both groups but there was a significant difference when comparing days of GnRH antagonist administration. The efficacy of the two starting protocols of the multiple dose GnRH antagonist evaluated was similar (10).

In a study by Lainas et al on patients with PCOS, they showed that the flexible GnRH antagonist protocol comparing to GnRH agonist, is associated with a similar ongoing pregnancy rate, lower incidence of OHSS, lower gonadotropin requirement and shorter duration of stimulation. They recommended flexible protocol as a treatment of choice for patients with PCOS undergoing IVF (4). 

Another systematic review of the RCTs, in which GnRH-agonist and GnRH- antagonist were compared ovarian stimulation outcomes for IVF in special patient groups, showed no differences in clinical outcomes. For PCOS patients, no differences in outcomes were found, except a significantly shorter duration of stimulation, in GnRH-antagonist multiple dose protocol. However, sample sizes was small and power to detect subtle differences was therefore limited (21).

To the best of our knowledge, our study was the first that compared standard and individualized GnRH antagonist protocol in one of the most important and debatable group of patients in ART cycles.

Both fixed and flexible GnRH antagonist protocols can be used in controlled ovarian stimulation for IVF/ICSI in patients with polycystic ovary syndrome. Our results sugesst that, it seems using flexible antagonist protocol in PCOS infertile patients is in favor of better outcomes in terms of number of good quality oocytes and embryo and possibility for cryopreservation for future cycles.
